# The effect of attentional bias modification on positive affect dynamics

**DOI:** 10.1038/s41598-024-74855-x

**Published:** 2024-10-09

**Authors:** Brage Kraft, Ragnhild Bø, Catherine J. Harmer, Nils Inge Landrø

**Affiliations:** 1 Division of Psychiatry, Diakonhjemmet Hospital, Postboks 23 Vinderen, 0319 Oslo, Norway; 2 Department of Behavioural Sciences, Oslo Metropolitan University, Oslo, Norway; 3 Department of Psychology, University of Oslo, Oslo, Norway; 4 Department of Psychiatry, Oxford University, Oxford, UK; 5 Oxford Health NHS Foundation Trust, Warneford Hospital, Oxford, UK

**Keywords:** Psychology, Depression

## Abstract

Negative attentional bias and alterations in positive affect dynamics constitute emotional vulnerability to depression. Attentional bias modification (ABM) aims to reduce emotional vulnerability to depression by fostering attentional deployment towards positive stimuli. In this randomized controlled trial, we examined whether ABM leads to changes in positive affect dynamics in a sample with an emotional vulnerability to depression (*N* = 65). Affect dynamics were measured based on experience sampling data gathered 14 days before and after ABM. During ABM, participants paid attention to pairs of emotional faces and responded to dots that were appearing in their place. There was an 87% chance for the dots to appear in place of the relatively more positive face, with the purpose to implicitly foster attentional focus on positive stimuli. In the control condition, there was a 50% chance of the dots to appear in place of the positive stimuli. Results showed that the lag-1 autocorrelation of affect (“inertia”) increased within the ABM group and decreased in the control group, but the findings were not robust and it was unclear whether ABM was the cause. There were no changes in the other affect dynamics indices. Improvements in depression during ABM were not associated with changes in affect dynamics, and affect dynamics post ABM did not predict depression symptoms six months later. In conclusion, the study showed no clear effect of ABM on affect dynamics.

Cognitive models assert that biases in attentional processes contribute to the development and maintenance of depression^1^. Selectively attending to negative stimuli has been emphasized as an important mechanism in reinforcing negative mood and maintenance of depression symptoms^2^. A meta-analysis confirms that depressed individuals, when compared to healthy subjects, have an attention bias toward negative stimuli^3^. A seminal study by MacLeod et al. showed that targeting this by experimentally manipulating attention away from negative stimuli using a modified dot probe task led to reduced emotional vulnerability, as indicated by increased susceptibility to feel distress when exposed to a stress manipulation^4^. Following this, researchers have aimed to modify attention bias in clinical groups.

Inspired by MacLeod et al.’s paradigm, attentional bias modification (ABM) aims to train attentional deployment towards positive stimuli^5–7^. ABM has shown promise in reducing emotional vulnerability^8^ and depressive symptoms^5,9–11^. Overall effect sizes, however, are small and its clinical relevance has been questioned^12,13^. Several short-comings with the current approach to ABM research have been identified^14^, and future development of ABM may be supported by more nuanced assessment of the potential underlying mechanisms^15^. One study, for example, using functional magnetic resonance imaging (fMRI), demonstrated that 14 days of ABM reduced activation of the amygdala and the anterior cingulate cortex when passively viewing negative images^16^. ABM may also have the potential to modulate the effects of affective stimuli on event-related potentials measured by electroencephalogram^17^ and can lead to reduced cortisol awakening response^5^. Impacts on self-reports of different trait-like emotion regulation processes known to increase the risk for depression, such as rumination^6^, have also been examined.

Notably, however, all previous ABM studies have in common that they assess “snap-shots” of emotional vulnerability as it manifests itself at the moment the participant is present in the lab, or by using self-report scales which may be limited by recall-bias. Future developments of the ABM research field may benefit from applying better measurement of depression and how we measure treatment response^18^. One promising way forward is to capture the real-time unfolding of depression symptoms over time using experience sampling methods (ESM)^18–20^. This approach focuses on subjects’ current mood state (or very recent ones), rather than asking for recall or summary over longer periods. Multiple assessments are conducted repeatedly, capturing how experiences and behavior vary over and across time^21^. Thus, ESM may enable the evaluation of moment-to-moment ebb and flow of affect, highlighting the dynamic nature of affective processing that is theorized to be central to the development and course of psychopathology^22^.

Three indeces of affect dynamics have been highlighted in the litterature^22^. Mean squared successive difference (MSSD) represents the magnitude of point-to-point change in affect and is often termed “instability”. The within-person standard deviation (SD) of affect observations represents the affective extremes and is often termed “variability”. The lag-1 autocorrelation (AC) of affect represents consistency from one time point to the next and is often termed “inertia”. Studies have showed that alterations in these dynamics may constitute emotional vulnerability to depression^19,22–26^, and have been associated with treatment response^19,27,28^. However, findings have been mixed. For example, depression has been linked with both increased^29,30^ and decreased^31,32^ AC.

In the present study, we attempted to move the ABM research field forward in line with recent theoretical approaches emphasizing depression as a dynamic phenomenon^18^. Although there have been studies examining the association between baseline attentional bias and affect dynamics (for example in anxious mood, see Iijima et al.^33^), no studies have examined whether ABM can produce changes in affect dynamics. We examined this in a sample with emotional vulnerability to depression, utilizing an interrupted ESM design where we measured affect during 14 days before and 14 days after ABM. Our analysis focused on positive affect because ABM aimed to modify attentional processing of positive stimuli, and because we have shown that ABM-related improvements in depression may be driven by changes in positive affect (i.e., interest)^34^. Finally, we examined whether changes in affect dynamics could explain ABM response (reductions in depression symptoms) and whether affect dynamics predicted depression symptoms six months later.

## Results

Table 1 presents demographic and baseline characteristics of the analyzed sample.

**Table 1:** Demographic and clinical characteristics of the final sample.

	ABM (*n* = 31)	Control (*n* = 34)
Sex, *n (%)*		
Female	20 (65%)	27 (79%)
Male	11 (35%)	7 (21%)
Age, *M (SD)*	44.6 (11.2)	42.9 (11.8)
Education level (ISCED), *M (SD)*	5.6 (1.6)	5.9 (1.2)
Depression status, *n (%)*		
Ongoing	14 (45%)	11 (32%)
Previous	31 (100%)	32 (94%)
Current SSRI, *n (%)*	12 (39%)	10 (29%)
Current comorbid anxiety disorder, *n (%)*	20 (68%)	23 (68%)
Baseline symptoms, *M (SD)*		
BDI	22.2 (10.8)	25.0 (10.4)
BAI	14.0 (9.7)	15.7 (8.7)
AUDIT	6.4 (4.6)	5.7 (6.1)
Baseline positive affect, *M (SD)*		
AC	0.31 (0.24)	0.39 (0.26)
MSSD	656 (503)	661 (531)
SD	31.0 (6.1)	31.2 (9.4)
Mean	70.9 (21.6)	67.7 (22.6)
ABM sessions completed, *M (SD)*	19.5 (6.9)	19.0 (6.6)
Days from ABM to six months follow-up, *M (SD)*	194 (13.7)	200 (31.8)

Note. ABM = Attentional bias modification; ISCED = International Standard Classification of Education; SSRI = Selective serotonin (and norepinephrine) reuptake inhibitors; BDI = Beck’s Depression Inventory; BAI = Beck’s Anxiety Inventory; AUDIT = Alcohol Use Disorders Identification Test; AC = lag-1 autocorrelation; MSSD = mean squared successive difference; SD = standard deviation.

### Effects of ABM on positive affect dynamics and mean positive affect

#### AC

 There was no effect of time (*b* =-0.40, *SE* = 0.22, *p* = .08) or ABM condition (*b* =-0.28, *SE* = 0.28, *p* = .32) on AC. There was a statistically significant effect of time by condition (*b* = 0.81, *SE* = 0.33, *p* = .02). AC increased in the ABM group (+ 0.10) and was reduced in the control group (-0.11). The changes within each group were not statistically significant (*p* = .09 and *p* = .07, respectively), and there was no statistically significant difference between the groups at post ABM (*p* = .09).

#### MSSD

There was no effect of time (*b* = -0.16, *SE* = 0.21, *p* = .43) or ABM condition (*b* = -0.01, *SE* = 0.25, *p* = .97) on MSSD. There was no effect of time by condition (*b* = 0.14, *SE* = 0.29, *p* = .63) on MSSD.

#### SD

There was no effect of time (*b* = -0.28, *SE* = 0.17, *p* = .10) or ABM condition (*b* = 0.02, *SE* = 0.25, *p* = .92) on SD. There was no effect of time by condition (*b* = 0.19, *SE* = 0.24, *p* = .44) on SD.

#### Mean affect

There was no effect of time (*b* = 0.04, *SE* = 0.11, *p* = .74) or ABM condition (*b* = 0.14, *SE* = 0.24, *p* = .57) on mean affect. There was no effect of time by condition (*b* = − 0.04, *SE* = 0.16, *p* = .79) on mean affect.

### Effects of positive affect dynamics on reduction in depressive symptoms

Overall, the sample demonstrated a reduction in depression symptoms from pre to post ABM (*M =* -5.5, *SD* = 7.6). Table 2 presents the results from the statistical models examining whether changes in affect dynamics can explain this response. In the first step, examining the role of time and MSSD, the passing of time predicted change in depression (*b* = -5.80, *SE* = 1.12, *p* < .001), but MSSD did not. This model explained 72% of the variance in depression change. In the second step, adding mean affect had a statistically significant contribution, and led to improved model fit, but only increased the explained variance by 1% point. In the third step, adding SD and AC led to reduced model fit, the explained variance reduced by 2% points, and there was no specific effect of affect dynamics. Follow up analyses examining the potential interaction between each of the affect dynamics indices and mean affect revealed no statistically significant interaction effects.

**Table 2:** Effects of positive affect dynamics on ABM response (reduction in depressive symptoms).

	b	SE	*p*-value
*MSSD (r*^*2*^ *=* 0.72, *AIC* = 833*)*			
MSSD	-0.86	0.84	0.31
*MSSD + mean affect (r*^*2*^ *= 0.73*, AIC = 633*)*			
MSSD	-0.12	0.88	0.89
Mean affect	-3.06	1.09	< 0.01
*MSSD + mean affect + SD + AC (r*^*2*^ *= 0.71*, AIC = 823*)*			
MSSD	1.74	1.64	0.29
Mean affect	-3.57	1.33	< 0.01
SD	-2.47	1.83	0.18
AC	-0.05	1.20	0.97

Note. ABM = attentional bias modification; MSSD = mean squared successive difference; SD = standard deviation; AC = lag-1 autocorrelation.

### Effects of positive affect dynamics on depression six months later

Multiple linear regression analysis (Table 3) showed no effect of affect dynamics variables post ABM or mean affect on depression symptoms six months after ABM, *F*(4, 52) = 0.19, *p* = .94, *r*^*2*^ *=* 0.01.

**Table 3:** Effects of affect dynamics and mean affect post ABM on depression symptoms at six months.

	b	SE	*p*-value
MSSD	-0.00	0.00	0.63
Mean affect	0.00	0.08	0.96
SD	0.05	0.23	0.84
AC	-8.77	10.52	0.41

Note. ABM = attentional bias modification; MSSD = mean squared successive difference; SD = standard deviation; AC = lag-1 autocorrelation.

## Discussion

The present study examined the effect of ABM on three indices of positive affect dynamics: MSSD, SD, and AC. Regarding AC, the ABM group and the control group demonstrated different AC trajectories; AC tended to increase in the ABM group and decrease in the control group. However, it is difficult to discern what this change means. Increasing AC may reflect that higher level of positive affect increasingly predicts higher level of positive affect, but can also reflect that lower level of positive affect increasingly predicts lower level of positive affect. In any case, the within-group differences from pre to post ABM (ABM: *p* = .09; control: *p* = .07) and the between-group differences post ABM (*p* = .09) were not statistically significant at the *p* < .05-level. Thus, we cannot conclude that ABM caused any changes in AC. Nevertheless, if the observed changes were more robust, a more parsimonious explanation would be that regression to the mean was the cause. Regarding the other affect dynamics indices, results showed that there was no effect of ABM on MSSD, SD, or mean affect.

Reduced depression symptoms across the groups from pre to post ABM were associated with increased mean positive affect but affect dynamics indices provided no added explanatory value. Moreover, affect dynamics did not predict depression symptoms six months after ABM. These findings line up with studies questioning the added benefits of assessing affect dynamics in explaining psychopathology^35^. It is possible that our current application of ESM was limited by an insufficient temporal resolution, an unreliable measure of positive affect, or a lack of consideration for contextual factors that influence affective experiences^35^. Future research should consider measuring affect on shorter timescales, improving the reliability of depression affect measures, and integrating context into ESM designs, such as linking affective responses to specific stressful life events that have been associated with depression (e.g., loss, social defeat)^36^. Deriving specific and testable hypotheses from established theories and considering the interval between assessments to match the timescale of the affective processes have also been highlighted as important steps forward^37^. Exploring non-linear relationships and employing machine learning techniques on raw emotional time series data may also be useful.

Our study has several strengths. It is the first to examine the effects of ABM on ESM-based measures. We used a well-established paradigm with a closely matched control condition, and randomization ensured control of any third variables. However, there are also limitations. The study aims and the statistical models were not preregistered, and the sample size was modest, leaving us questioning whether a another data set with a larger sample could provide more conclusive results. The measure of positive affect was based on only two items and not formally validated. Future studies should use broader and validated measures of positive affect, for example using the Positive and Negative Affective Schedule^38^. Comparing the ABM condition with other types of control conditions could be also be more fruitful when investigating the potential clinical effectiveness of ABM (as discussed by Blackwell et al.^39^). Finally, our study may be limited by the main study showing no specific ABM effect on overall symptom severity^6^.

In conclusion, ABM did not lead to clear changes in positive affect dynamics, and affect dynamics post ABM did not predict depression symptoms six months later. Future studies, providing a larger sample, or leveraging other types of analytical approaches (for example based on the network approach^40–42^), may yield other results.

## Methods

### Sample

The present study was based on data from a randomized controlled trial (*N* = 101) examining the potential of ABM in reducing depression symptoms^6^. Individuals reporting depressive complaints were recruited by community and social media advertisement. Inclusion criteria were previous or current Major Depressive Disorder, age 18–65 years, and fluency in Norwegian. Exclusion criteria were manic episodes, psychosis, and neurological disorders. Diagnostic status was assessed at the lab using the *MINI International Neuropsychiatric Interview*^43^. All participants provided written informed consent, and the study was performed in accordance with the Declaration of Helsinki. The study and the experimental protocol were approved by the Regional Committee for Medical and Health Research Ethics in Norway (no. 2019/330) and the Norwegian Social Science Data Services.

### Clinical assessment

Depression symptoms were assessed using the Beck’s Depression Inventory-II (BDI)^44^. We also report on anxiety symptoms using Beck’s Anxiety Inventory (BAI)^45^ and self-reported alcohol consumption habits (AUDIT)^46^.

### ESM

ESM data was collected using the PsyMate app^47^ installed on participants’ personal smartphones. Participants received in-person instruction on how to use the app. The app notified participants five times per day at random intervals between 8.30 AM and 10.30 PM (total number of measurements = 70). At each measurement participants were asked to complete a short questionnaire introduced by the sentence: “How have you been the last hour?” (see Supplementary Materials). The questionnaire consisted of several items measuring depression symptoms using a slider scale with values going from 0 (not at all) to 100 (very much). Participants were instructed to use approximately one minute to answer the whole questionnaire, and the questionnaire had to be completed within 30 min (or else a non-response was recorded). This study reports on two items measuring positive affect. The positive affect items were as follows: interest (“How interested have you been in what you have been doing?”) and happiness (“How happy have you been?”). Interest and happiness were moderately correlated within each time point pre (*r* = .56, *p* < .001) and post ABM (*r* = .58, *p*  < 0.001). A compound score of positive affect was calculated by taking the sum of the items.

#### Affect dynamics indices

Affect dynamics indices were calculated in R Statistical Software (version 4.4.1^48^) in line with the approach described by Funkhouser et al. with the goal to maximize the reliability of the estimates^25^. A missing beep was added at the end of each day to avoid estimating night-to-morning lags. Participants who had less than 10 surveys were excluded. The remaining participants completed on average 37 surveys (53%) pre ABM (*n =* 74; *M* lag = 179 min, range = 25–362 min) and 35 surveys (50%) post ABM (*n* = 57; *M* lag = 176 min, range = 14–357 min). The lag-1 autocorrelation (AC) of positive affect, representing the degree of affect consistency over time, was calculated using the autoR function in the *psych* R package (version 2.4.3^49^) after excluding participants who had < 10 consecutive completed surveys (pre: *n* = 57; post: *n =* 40). Mean squared successive difference (MSSD) represents the average magnitude of lag-to-lag shifts in affect intensity, and was calculated using the mssd function in the *psych* R package^49^ after excluding lags +/- 1.5 *SDs*. The standard deviation of affect (SD), representing the extent to which the subject used wider range of the scale to rate their affect, was calculated by taking the standard deviation of affect within each subject. Mean affect was calculated by taking the mean affect ratings within each subject.

### ABM

**Fig. 1:**
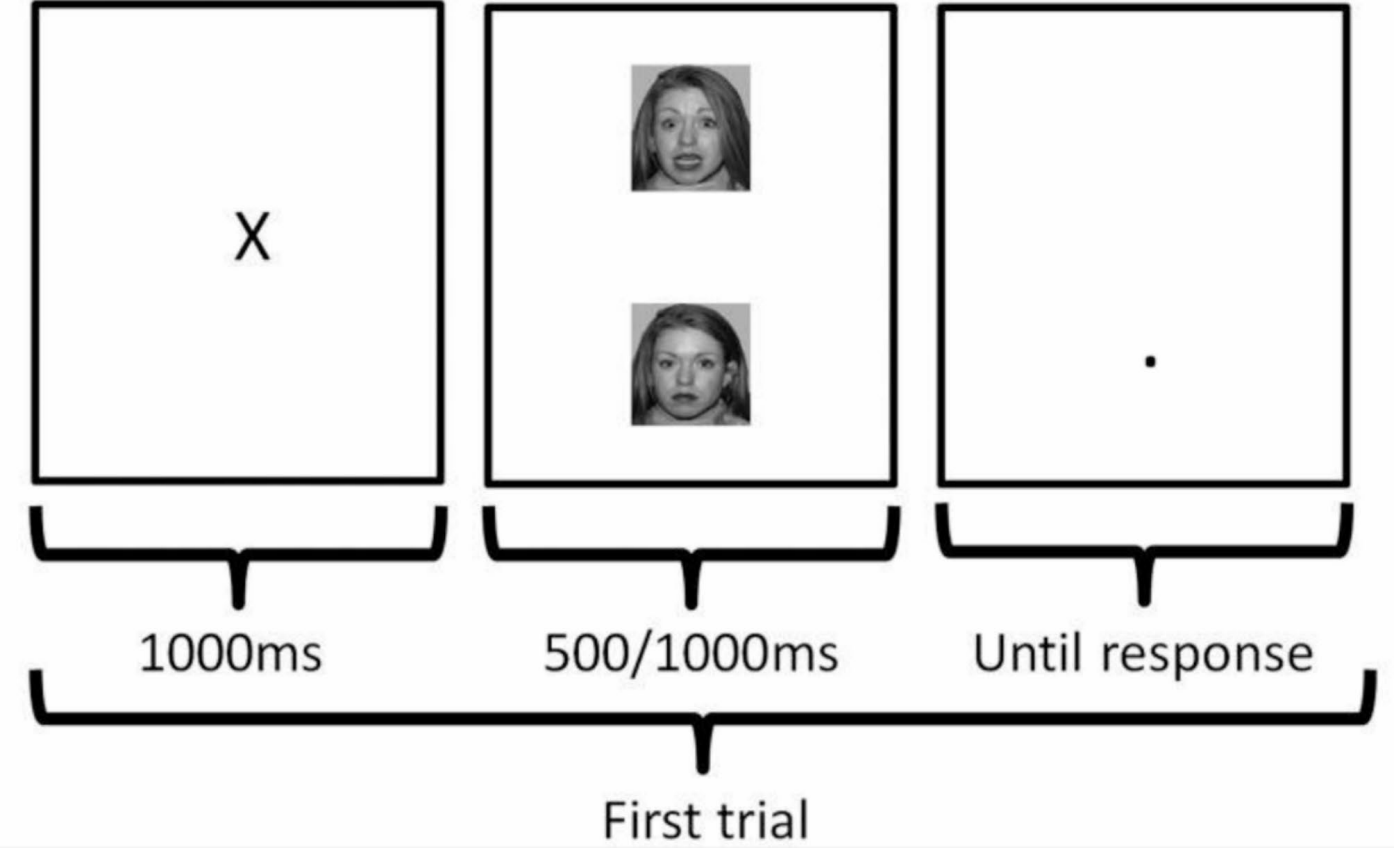
The ABM positive contingency condition. Adapted from Browning et al.^5^ (CC BY 4.0).

ABM consisted of 28 sessions of a computerized visual dot-probe procedure for 14 consecutive days (i.e., twice daily), adapted from Browning et al.^5^ and identical to the one reported in Jonassen et al.^7^. Each session lasted for circa 5–7 min and was conducted on laptops provided to the participants. Stimuli were pairs of images of faces displaying positive (happy), negative (fear or anger) and neutral faces (see Fig. 1). Each stimuli pair was displayed horizontally in random order, for either 500 or 1000 ms, and consisted of one out of three valence pairs (positive-negative, negative-neutral, positive-neutral). Immediately after stimuli presentation, a probe (one or two dots) appeared in one of the locations of the previously displayed stimuli. Participants were asked to indicate the correct number of dots in the probe as fast and accurately as possible. One session consisted of 96 trials with equal number of the three stimuli pairs.

There were two conditions. In the positive contingency condition, the probe appeared in the location of the screen where the relatively more positive stimuli were displayed for 87% of the trials. The rationale for this contingency is that participants should implicitly learn to attend to the relatively more positive stimuli. In the no-contingency (control) condition there was no contingency between probe location and stimuli (i.e., only 50% of the probes appeared at the location of the relatively more positive stimuli).

### Procedure

After diagnostic assessment and inclusion in the study, participants were enrolled to 14 days of ESM and carried out their daily life as normal. Participants then returned to the lab for additional clinical assessment (using self-reports) and initiation of ABM. Allocation to condition (positive contingency vs. control) was determined by an independent lab technician by lottery draw from slips in a container (1:1 ratio), and participants and study investigators were blinded to treatment allocation. After ABM, participants returned to the lab for clinical assessments, and then started a new 14-day ESM assessment. A final clinical assessment was conducted online six months after the intervention.

### Statistical analyses

**Fig. 2:**
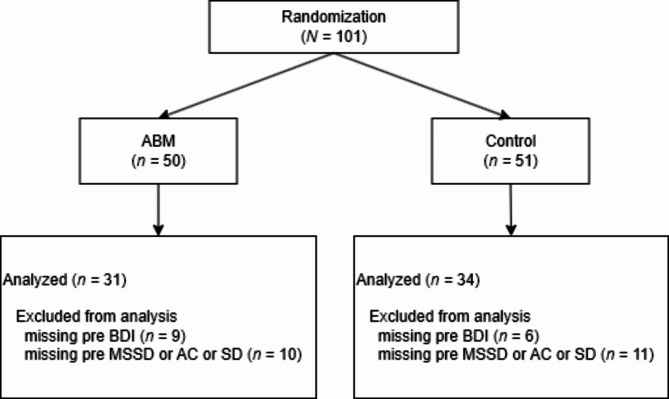
Flow diagram of sample. ABM = attentional bias modification; BDI = Beck’s Depression Inventory II; MSSD = mean squared successive difference; AC = lag-1 autocorrelation; SD = standard deviation.

We analyzed data from participants who provided data on depression symptoms and at least one of the affect dynamics indices pre ABM (*N* = 65, see Fig. 2). The data were analyzed in R Statistical Software (version 4.4.1^48^).

First, we examined the effect of ABM on affect dynamics change (dependent variables: AC, MSSD, SD) in three separate linear mixed-models. Linear mixed-model analysis is suitable for analyzing repeated measurements data as it can estimate both fixed effects (across all observations) and random effects (accounting for variability between subjects). The fixed factors were time (pre, post) and ABM condition (positive contingency, control), and the models included random intercepts. Missing variables post ABM (BDI: ABM = 4, control = 3; MSSD/SD/mean affect: ABM = 5, control = 8; AC: ABM = 12, control = 14) were not imputed, as mixed-model analysis without ad hoc imputation is more powerful compared to other alternatives^50^. Models were estimated using restricted maximum likelihood in the *lme4* R package (version 1.1–35.3^51^) and interaction effects (time x condition) were evaluated based on least squares means calculated using the *lmerTest* R package (version 3.1.3^52^).

Second, also using mixed-model analysis, we examined the potential role of affect dynamics changes in explaining response to ABM (changes in depression). When benchmarked against mean affect levels, studies have shown that affect dynamics indices provide no added incremental value in predicting well-being^35^ or depression^19^. We therefore specified a model with depression as the dependent variable, and time and MSSD as the independent variable before iteratively adding mean affect, SD, and AC (in line with Dejonckheere et al.^35^ and Bosely et al.^19^).

Finally, using multiple linear regression, we examined the role of affect dynamics and mean affect post ABM in predicting depression symptoms six months later. The predictors entered in the model were MSSD, mean, SD, and AC at post ABM, and the outcome variable was depression symptoms at six months. Any missing independent variables (MSSD/SD/mean affect = 13; AC = 26) were handled by imputing the mean.

## Supplementary Information

Supplementary Information.

## Data Availability

The dataset analyzed during the current study is not publicly available due to privacy reasons but is available from the corresponding author on reasonable request and with necessary ethical approval.
