# Supplementary material for: The effect of attentional bias modification on positive affect dynamics
Source: Sci Rep. 2024 Oct 9;14:23628. doi: 10.1038/s41598-024-74855-x (PMC11464596; doi:10.1038/s41598-024-74855-x)
Supplement: Supplementary file 1 — Supplementary Information. [file 41598_2024_74855_MOESM1_ESM.docx]

**Supplementary Materials for
Kraft et al. (2024). *The Effect of Attentional Bias Modification on Positive Affect Dynamics***

**Experience sampling methods affect questionnaire**

Participants responded using a slider scale with values going from 0 (not at all) to 100 (very much). The items were as follows:

1. How sad have you been? (sadness)
2. How tired have you been? (fatigue)
3. How interested have you been in what you have been doing? (interest)
4. How happy have you been? (happiness)
5. How great difficulties have you had concentrating? (concentration problems)
6. How much have you been ruminating? (rumination)
7. How active have you been (physically/mentally/socially)? (activity)
